# Therapeutic Notes

**Published:** 1914-03

**Authors:** J. M. Fortescue-Brickdale


### Therapeutic Notes.

### COLLOID METALS.

THE term " colloid," originally applied to a certain group of substances owing to their uncrystallisable, glue-like nature, has gradually changed its connotation, and is now used to describe a certain condition of matter rather than a particular class of bodies. By using appropriate methods, many substances have been obtained in the colloid state, that is to say, in the form of fine particles, which can be suspended in a fluid medium and which have certain definite physical properties. In the present short note we may consider in order (1) the preparation and physico-chemical properties of colloid solutions, (2) their physiological action, (3) their therapeutic uses.

1. If two bodies, which, in the presence of traces of an electrolyte produce a precipitate, are allowed to act on one another in the absence of the electrolyte, a colloidal solution results, and no precipitate is formed. Slow reduction processes also result in the formation of colloid metals. In contradistinction to these chemical methods there is the physical method first employed by Bredig, which consists of passing an electric current through water from metal terminals. A colloid solution of the metal used results.

Colloid solutions stand midway between an ordinary suspension and a true molecular solution. The particles of the colloid are small enough to pass through an ordinary filter, but large enough to be visible by means of the ultramicroscope. They vary from $0.1\mu$ to $0.001\mu$ in diameter, and are in a state of perpetual motion, oscillatory or Brownian in the larger particles and translational also in the finer ones. These particles have positive or negative electrical charges which vary with different substances and may be reversed by altering the solvent. Traces of an electrolyte will throw down some colloid solutions at once, provided that the ions have a contrary electric sign to the colloid particles. Another class of colloid is not so easily precipitated, and can be brought again into solution by removing the electrolyte. These are known as "reversible" colloids, and include such bodies as protein, gelatin, etc. Traces of a reversible colloid will prevent the precipitating action of an electrolyte on one of the former class. There is, of course, an immense aggregate of surface presented to the fluid medium by the colloidal particles. This surface will vary inversely as the fineness of the particles; in fine colloidal solutions there is a very great surface tension between the particles and the solvent and a large storage of surface energy. The colloids also have great "adsorptive" powers.

2. Colloidal solutions of metals inhibit the growth of bacteria. The cause of this does not seem quite clear. It is no doubt in some way dependent on the physical condition of the particles, but not entirely, for colloidal solutions of some metals seem to be more actively antiseptic than others. When injected into the animal body certain metabolic changes have been observed, such as increased elimination of urea and uric acid, and a rise in the respiratory quotient. There is also a variable degree of leucocytosis, principally of the mononuclear cells, which is not usually well marked in healthy persons. A temporary rise in arterial blood-pressure and a slight pyrexia have also been noted. The opsonic index is raised apparently by activation of the blood serum. In sick persons there is apt to be a more severe reaction with a considerable rise of temperature, cyanosis and a rapid pulse. It has been ascribed to the setting free of morbid products by the destruction of a large number of bacteria or cells. The leucocytosis is said to be very well marked in those diseases in which it is a normal reactionary process.

3. Both electrically and chemically prepared colloids have been used in medicine, and although the latter have been said by Robin and others of the French school to be inactive and useless, there does not seem at present to be much clinical evidence in support of this statement. Naturally these solutions have mainly been employed in conditions of general septicæmia or local bacterial infection. Among the latter inflammation of the mucous membranes of the nose and throat have been successfully treated by the application of colloidal *silver*, which has also been injected directly into the pleura, the bladder and even solid organs like the epididymis. Colloidal *mercury* has been employed for syphilitic affections locally and generally, and *selenium* and *rhodium* in general infections and cancer. In the latter disease softening of inoperable secondary deposits is said to have occurred after intravenous injections. Colloidal *iron* has long been employed as Liquor Ferri Dialyzatus, which is a colloidal solution of Ferric Hydroxide. The metal itself has recently been prepared in colloidal form and injected intravenously in cases of chlorosis and secondary anæmia. Robin has used colloidal *sulphur* in chronic or subacute rheumatoid conditions. The hydroxide of *copper* has been used in cancer, and an electrically-prepared colloid of the metal in tuberculosis. In the latter disease the injections must be intravenous, and probably only ameliorate secondary infections.

One or two cubic centimetres (fifteen to thirty minims) of the solution is the usual dosage, but most manufacturers supply sealed ampoules containing one dose each for intravenous injections. The solutions should not be boiled, and the electrically-prepared colloids should not be rendered isotonic with sodium chloride unless previously "protected" by the presence of some reversible colloid. Good results are claimed by responsible observers for this treatment, especially in septicæmia, but it must be remembered that successes are more often recorded than failures, and that even when the latter are published in the medical press the reports are not assiduously circulated by the manufacturers among the members of the profession.

J. M. FORTESCUE-BRICKDALE.

